# Pain Management Following Intracavitary Brachytherapy Procedures: A Prospective, Randomized, Double-Blind Study

**DOI:** 10.7759/cureus.67681

**Published:** 2024-08-24

**Authors:** Jagdeep Sharma, Harsimran Walia, Lalita G Mitra

**Affiliations:** 1 Anaesthesia, Critical Care, and Pain Medicine, Homi Bhabha Cancer Hospital and Research Centre, New Chandigarh, IND; 2 Anaesthesia, Critical Care, and Pain Medicine, Homi Bhabha Cancer Hospital and Research Center, New Chandigarh, IND

**Keywords:** pain, day-case procedures, spinal anesthesia, brachytherapy, fentanyl, dexmedetomidine

## Abstract

Background and objectives

Intracavitary applicators are a source of significant discomfort after brachytherapy procedures while undergoing subsequent radiation treatment. With strides towards opioid-sparing anesthesia and analgesia, it’s essential to find appropriate substitutes. This procedure requires adequate relaxation of pelvic muscles during the procedure and proper analgesia after the procedure, with the presence of intracavitary applicators, needed for radiation treatment. We studied the day-case safety and analgesic efficacy of adjuvants dexmedetomidine 3 µg and fentanyl 15 µg intrathecally to low-dose 0.5% hyperbaric bupivacaine.

Methods

Seventy females scheduled for brachytherapy procedures were randomly allocated to receive either Group I (0.5% hyperbaric bupivacaine (1.8 ml) plus 3µg dexmedetomidine (0.3ml)) or Group II (0.5% hyperbaric bupivacaine (1.8 ml) plus 15µg fentanyl (0.3ml)). The primary outcome was to assess and compare the brachytherapy (day-case) feasibility with 3µg dexmedetomidine and 15µg fentanyl (time taken to meet hospital discharge criteria). The secondary outcomes were the absolute duration of spinal analgesia, pain scores, patient satisfaction scores, and any associated adverse events. Data analysis was done using IBM SPSS software for Windows, version 21.0 (IBM Corp., Armonk, NY).

Results

All patients in Group I were discharged on the same day without any adverse effects. They underwent an intracavitary brachytherapy procedure under spinal anesthesia with stable hemodynamics successfully. The mean time taken to meet hospital discharge criteria in Group II was shorter than in Group I (258.43 ± 27.460 vs. 335.71 ± 21.114). The mean absolute duration of spinal analgesia was significantly longer in Group I as compared to Group II (406.82 ± 51.78 mins vs. 267 ± 16.23 mins) (p<.001). Seventeen patients required rescue analgesia in Group II versus eight in Group I (p<0.025).

Conclusion

Patients in both groups received excellent analgesia with enhanced patient satisfaction. Three µg intrathecal dexmedetomidine as an adjuvant to low-dose hyperbaric bupivacaine can be used safely in day-case brachytherapy procedures. It provides adequate anesthesia and prolonged spinal analgesia as compared to 15 µg fentanyl.

## Introduction

Adequate pain management is essential for patients after brachytherapy procedures. Various adjuvants to local anesthetics have been used to improve pain management and enhance overall satisfaction. Opioid-sparing anesthesia offers particular advantages in opioid-tolerant patients, such as patients with cancer-related pain, chronic pain, and opioid addiction [1]. Increased use of opioids in cancer patients further compromises the immune system by decreasing the progenitor cells of lymphocytes and macrophages. It also suppresses the T cell-mediated immunity [2]. Day-case procedure rates have steadily increased across the world. Development in medical technology, surgical skills, the advent of new anesthetic agents, techniques, and improved methods of analgesia have facilitated this [3]. Intracavitary brachytherapy has acquired an essential role in treating carcinoma of the cervix and presents numerous challenges. It is excruciating and requires good immobilization and analgesia. It is usually performed as a day-case procedure. In the UK, 'ambulatory surgery' refers to patients being discharged from the hospital shortly after surgery; in the USA, this term may also apply to admissions for up to 23 hours. Low-dose (<10 mg) intrathecal bupivacaine is associated with a shorter time to void and discharge home, although a few patients may still have a long recovery time. Spinal anesthesia can be suitably performed for various day-stay (ambulatory) surgical procedures [4]. Spinal anesthesia administered with a low dose of local anesthetics and adjuncts provides a superior recovery profile.

Various adjuvants have been used to avoid intraoperative visceral and somatic pain and to provide prolonged postoperative analgesia. These include opioids, alpha-2 (α2) agonists, neostigmine, vasoconstrictors, etc. Clonidine and dexmedetomidine are two α2 agonists affecting pre- and postsynaptic α2 receptors [5]. Fentanyl is the most commonly used short-acting opioid intrathecally. Intrathecal opioids prolong the duration of anesthesia and analgesia, along with side effects such as itching, urinary retention, nausea, vomiting, and respiratory depression [6].

The role of 15 µg fentanyl has already been established for day-case intracavitary brachytherapy procedures by previous studies. Dexmedetomidine, a highly selective α2 adrenergic agonist, is also emerging as a valuable adjunct to regional anesthesia and analgesia, where gradually evolving studies can build the evidence for its safe use in central neuraxial blocks [7]. It prolongs the sensory block by depressing the release of C fiber transmitters and hyperpolarizing postsynaptic dorsal horn neurons [8-9].

No study to date has tried to establish the day case feasibility with 3 µg dexmedetomidine and also compared it with fentanyl 15 µg as adjuvants to low-dose hyperbaric bupivacaine in patients undergoing intra-cavitary brachytherapy procedures. The primary outcome was to assess the day-case feasibility of intrathecal 3 µg dexmedetomidine as an adjuvant to low-dose hyperbaric bupivacaine in day-case intracavitary brachytherapy procedures.

The secondary outcomes of our study were to compare the absolute duration of spinal analgesia, pain scores, time taken to meet hospital discharge criteria, and any associated adverse effects with the use of both adjuvants. We hypothesized that 3 µg dexmedetomidine can be used safely in day-case procedures and provide a prolonged duration of spinal analgesia than fentanyl 15 µg.

## Materials and methods

After obtaining due approval from the Institutional Ethical Committee of Homi Bhabha Cancer Hospital and Research Centre, New Chandigarh, Punjab, India, we conducted the interventional, randomized, parallel-group active-controlled, double-blind trial comparing the anesthetic and analgesic efficacy of adjuvant dexmedetomidine (3 µg) with fentanyl (15 µg) intrathecally to low-dose hyperbaric bupivacaine in day-case intracavitary brachytherapy procedures. The Clinical Trials Registry-India (CTRI) registration number of this trial is CTRI/2022/05/042677. The patients were enrolled from June 2022 to November 2022, after which the trial was closed owing to the achievement of enrolment of the total number of patients as derived from the sample size calculation. The sample size was based on a study by Rahimzadeh et al. [10] and showed a power of 0.9, a type-1 error of 0.05, and a confidence interval of 95%, allowing a 10% dropout rate. We conducted the study following the ethical guidelines of the Declaration of Helsinki.

**Figure 1:**
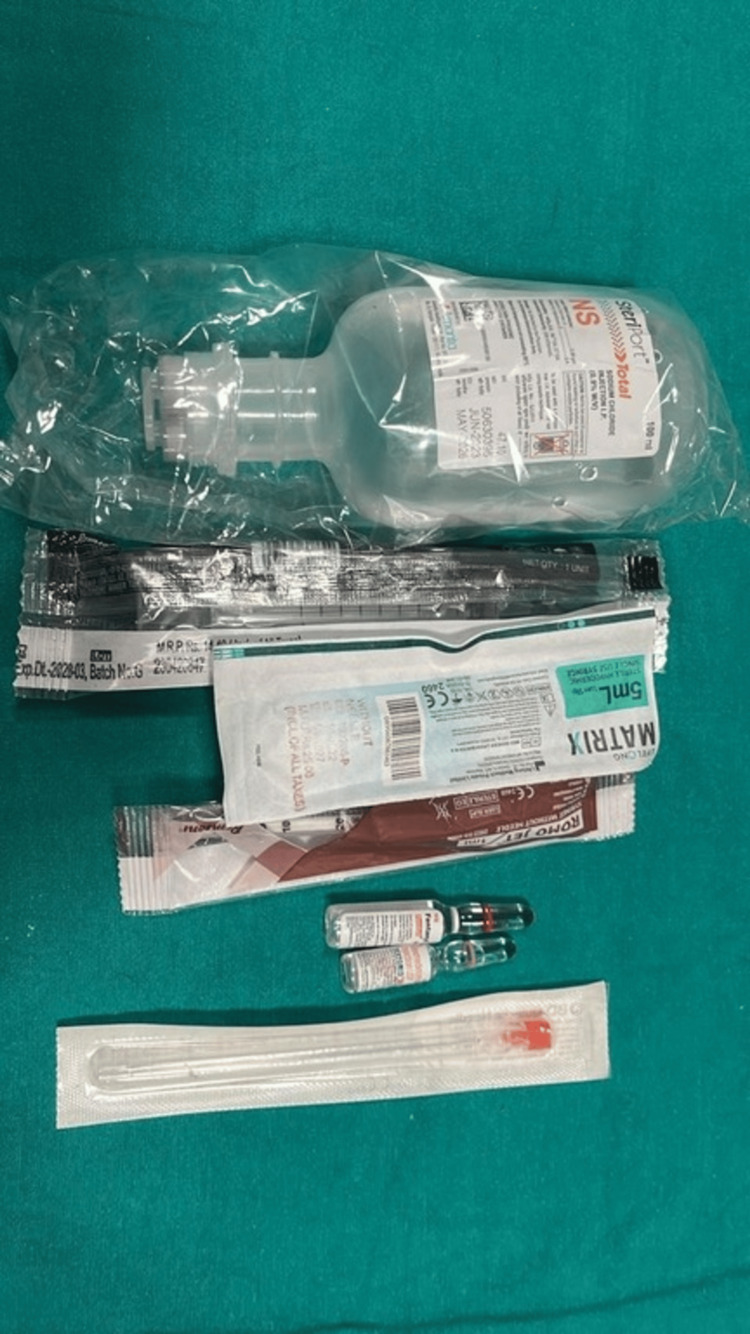
Equipment used during the procedure Quincke’s spinal needle, dexmedetomidine ampoule, fentanyl ampoule, insulin syringe, and normal saline (NS) bottle

Seventy patients were randomly assigned to Group I or Group II using a computer-generated table of random numbers. Sequentially numbered, sealed opaque envelopes were used for group allocation. Group I patients received dexmedetomidine 3 µg (0.3 ml) with 9 mg (1.8 ml) of hyperbaric bupivacaine (total volume 2.1 ml). In contrast, patients in Group II received fentanyl 15 µg (0.3 ml) with 9 mg (1.8 ml) of hyperbaric bupivacaine (total volume 2.1 ml). A different anesthesiologist filled and injected the drugs to reduce subjective and objective bias. This is to emphasize that this is a double-blind trial. American Society of Anesthesiologists (ASA) grades I and II patients aged 18 to 75 years posted for intracavitary brachytherapy were included in this study. Written informed consent was taken. Patients who refused spinal anesthesia, required admission overnight, with contraindications to spinal anesthesia (including coagulopathy, low absolute neutrophil count, and being allergic to local anesthetic), and patients with raised intracranial pressure, peripheral neuropathy, demyelinating central nervous system disorders, and local sepsis were excluded. The equipment used during the brachytherapy procedure is shown in Figure 1.

A thorough preoperative evaluation was done a day before the procedure. Patients were kept nil per oral for six hours for solids and two hours for clear liquids before the start of the procedure. On arrival of the patient in the operation theater, all standard ASA monitors, including electrocardiography, pulse oximetry, and non-invasive blood pressure, were attached. Baseline vitals were recorded, and the intravenous cannula was secured. Patients were preloaded with 5 ml/kg of Ringer's lactate. Under aseptic precautions, spinal anesthesia was given at the level of L3-L4 interspace in a sitting position using a midline or paramedian approach by a 25 G Quincke spinal needle. Patients were made supine following the block. The appropriate head-down position was given till the level of anesthesia reached the T-10 position. The level of the sensory block was checked bilaterally by the pinprick method with a 23-gauge hypodermic blunt needle. The onset of sensory block was assessed from the time of injecting the drug into the subarachnoid space till complete analgesia at the level of T-10 was assessed by sterile pinprick every two minutes. The duration of the sensory block was defined as the time of regression of two segments in the maximum block height, evaluated by pinprick. The degree of motor blockade was assessed using a Modified Bromage Scale [11]. The Campbell sedation score was used for assessing the degree of sedation and scoring [12].

The procedure was allowed to commence on achieving adequate sensory block height at T-10. Hemodynamic parameters were recorded before, at the time of intrathecal injection, five, 10, 15, 20, 25, and 30 minutes after, and subsequently every 15 minutes till completion of the procedure. The Visual Analog Scale (VAS) was used for assessing pain scores and was rated from one to 10. Baseline pain scores were evaluated five minutes before intrathecal injection and subsequently, every 30 minutes till the procedure was over. The incidence of sedation, pruritus, nausea, and vomiting was also recorded. All durations were calculated considering the time of spinal injection as the starting time. Continuous monitoring of the hemodynamic parameters was done. Hypotension with a decrease in systolic blood pressure (SBP) by 30% from baseline was treated with IV boluses of 6 mg mephentermine and crystalloids. Bradycardia was defined as a heart rate < 50/min and was managed with an injection (inj) of atropine 0.6 mg IV stat. All patients were monitored post procedure for 12 hours.

Postoperatively, the pain scores were recorded every 30 minutes with VAS for 12 hours. The absolute duration of spinal analgesia was defined as the period from intrathecal injection of the drug to the first requirement of rescue analgesia (VAS ≥ 4) by the patient in the postoperative period. Rescue analgesia was given in the form of an injection of paracetamol 15 mg/kg if VAS ≥ 4. The time taken for a complete reversal of the Modified Bromage Score to zero was also noted. Day-case feasibility depended upon the safe discharge of patients on the same day without any urinary retention or sedation. They were deemed fit for discharge after voiding and meeting the hospital discharge criteria. The time taken to meet hospital discharge was assessed and compared in both groups. Patient satisfaction score rated on VAS, including overall quality of anesthesia and postoperative analgesia, was assessed 12 hours after the procedure.

Data entry and analysis were done using IBM SPSS software for Windows, version 21.0 (IBM Corp., Armonk, NY). Categorical variables were represented as frequency and percentage, and continuous variables were represented as mean ± standard deviation. The Shapiro-Wilk test was used to check for the normality of data. Categorical variables were analyzed using Pearson's chi-squared test/Fisher's exact test, and continuous variables were analyzed using an independent sample unpaired t-test/Mann-Whitney U test (Wilcoxon rank sum). The significance of the p-value was taken as p < 0.05.

## Results

**Figure 2:**
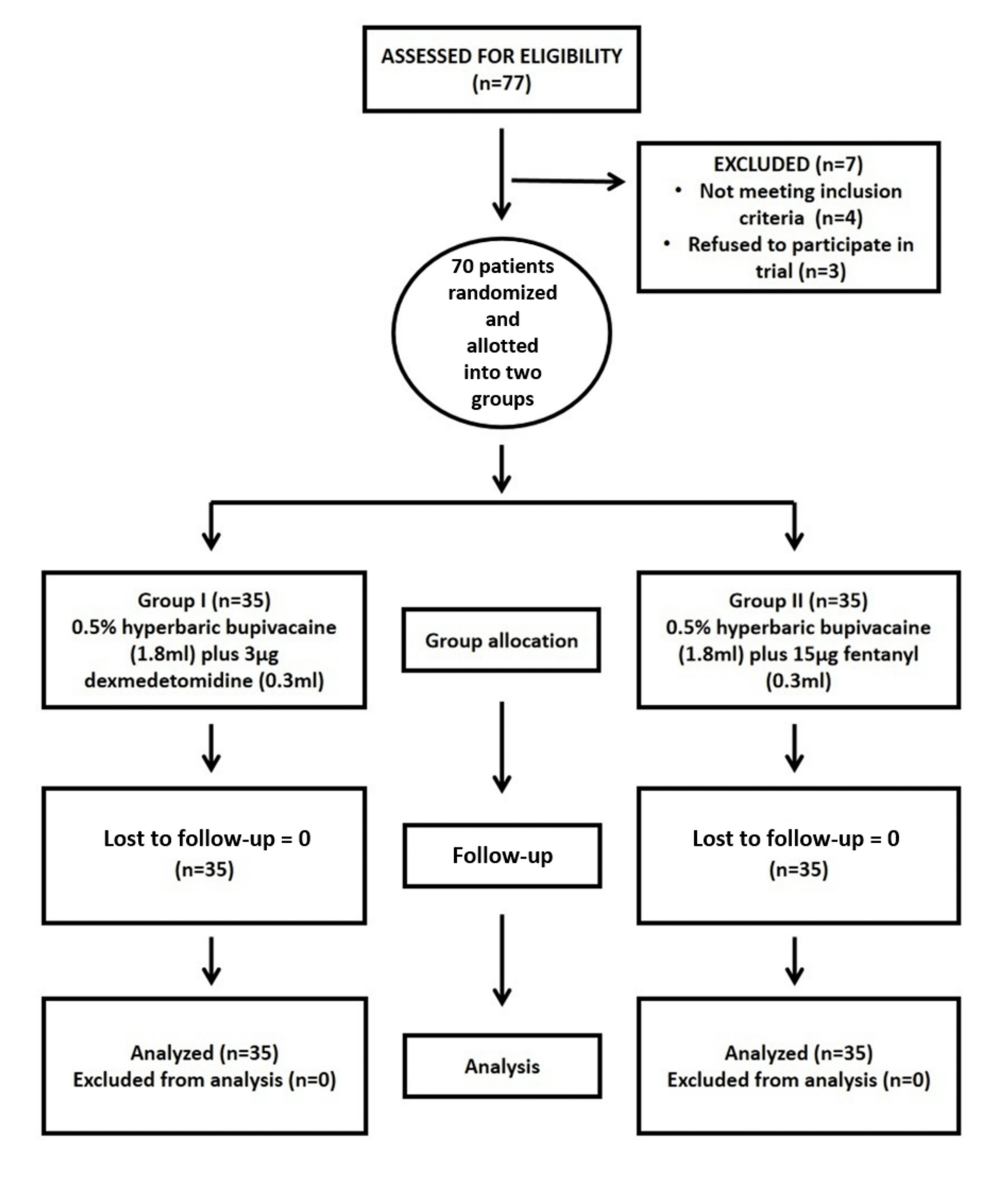
The CONSORT diagram outlining the study process CONSORT: Consolidated Standards of Reporting Trials

The Consolidated Standards of Reporting Trials (CONSORT) diagram which outlines the flow of the study is depicted in Figure 2.

Our study evaluated the outcomes of intracavitary brachytherapy in two groups of patients (referred to as Group I and Group II). All the patients in Group I were discharged the same day with no residual weakness or sedation. The procedure was performed successfully on all patients without any complications or adverse events, with stable hemodynamic parameters. The difference in demographic profiles and preoperative hemodynamic vitals was comparable and statistically insignificant between the two groups (Table 1).

**Table 1:** Demographic data of the patients in both groups

	Group I (n = 35)		Group II (n = 35)	
	Mean	± SD	Mean	± SD
Age (years)	52.63	9.046	53.57	9.017
Weight (kg)	60.03	10.360	56.40	7.232
Height (cm)	156.80	3.151	155.97	3.121
Duration of the procedure (minutes)	37.29	2.803	37.00	4.058

There was no pain and associated sedation during the procedure in both groups. There was no sedation in the post-procedure period. The time taken for the sensory block to reach the T-10 dermatomal level was similar in both groups, indicating comparable onset times. The time for a two-segment sensory regression was also similar between the groups. The mean time for the sensory block to reach the T-10 level was found to be 6.95 ± 0.73 minutes in Group I as compared to 6.90 ± 0.71 minutes in Group II (p > 0.05). The mean time for two-segment sensory regression was 63.86 ± 4.86 mins in Group I and 62.57 ± 7.21 mins in Group II (p > 0.05).

However, there were some notable differences between the groups. The VAS scale was used to assess the pain scores. If VAS ≥ 4, inj paracetamol 1 gram was administered intravenously and used as a rescue analgesic. The patients did not complain of any pain during the procedure. For the first five hours post procedure, the mean pain scores in Group I were significantly lower than in Group II. The pain scores at the time of discharge were comparable in both groups. A greater number of patients in Group II required rescue analgesic doses as compared to Group I. The absolute duration of spinal analgesia was significantly longer in Group I as compared to Group II (406.82 ± 51.78 minutes vs. 267 ± 16.23 minutes) (p<.001). The time taken for a complete reversal of the Modified Bromage Score was 161.29 ± 14.56 minutes in Group I and 102.22 ± 11.00 minutes in Group II (p<0.001). The time taken to void and subsequently meet hospital discharge criteria was significantly shorter in Group II as compared to Group I (258.43±27.46 minutes vs. 355.71 ± 21.11 minutes; p<.001) (Table 2).

**Table 2:** Absolute duration of spinal analgesia, time to zero modified Bromage score, and time taken to meet hospital discharge criteria of patients in both groups Student's t-test unpaired; not significant (NS): p > 0.05; *p<0.05 significant

	Group	N	Mean ± SD	P-value
Time taken to meet hospital discharge criteria (minutes)	Group I	35	320.70 ± 29.00	<0.001^*^
	Group II	35	254.45 ± 22.40	
Absolute duration of spinal analgesia (minutes)	Group I	35	406.82 ± 51.78	<0.001^*^
	Group II	35	267.00 ± 16.23	
Time to zero Modified Bromage Score (minutes)	Group I	35	161.29 ± 14.56	<0.001^*^
	Group II	35	102.00 ± 10.99	

There was a significant difference in the number of patients requiring rescue analgesia; 17 patients required rescue analgesia in Group II versus eight patients in Group I (p = 0.025) (Table 3).

**Table 3:** Number of patients requiring rescue analgesia before discharge in both groups

Number of patients requiring rescue analgesia before discharge	Group I (n = 35)		Group II (n = 35)		Total
	N	%	N	%	
No	27	77.1	18	51.4	45
Yes	8	22.9	17	48.6	25
Total	35	100.0	35	100.0	70

All patients in the dexmedetomidine group were discharged the same day without any associated adverse effects. Although patient satisfaction scores did not show a statistically significant difference between the two groups, a higher number of patients in Group II reported experiencing nausea or vomiting compared to Group I.

## Discussion

The presence of intracavitary applicators for radiation treatment requires adequate and effective analgesia post procedure. The intracavitary applicators stay in situ for approximately two to three hours. With giant strides towards opioid-sparing analgesia, it’s also essential to find appropriate substitutes. The role of 15 µg fentanyl as an adjuvant to low-dose hyperbaric bupivacaine in intracavitary brachytherapy procedures has already been established by previous studies. Various studies have already established the better quality and prolonged analgesia provided by dexmedetomidine over fentanyl.

Nethra et al. failed to establish the day-case safety of 5 µg dexmedetomidine as an adjuvant to 6 mg of low-dose hyperbaric bupivacaine in ambulatory perianal surgeries. They studied the duration of analgesia and sensory and motor block characteristics for ambulatory perianal surgeries. The time taken to void was 422.30 ± 87.59 minutes. This time to void can be attributed to the 5µg dose of intrathecal dexmedetomidine [13].

Bi et al. established the safe use of 3 µg dexmedetomidine as an adjuvant to ropivacaine by investigating its use in a high-risk obstetric subset of patients. The patients underwent the procedure successfully. The time for complete reversal of the motor block was 2.24 hours with 3 µg dexmedetomidine. They concluded that 3 µg intrathecal dexmedetomidine as an adjuvant improved intraoperative somato-visceral sensory block characteristics and postoperative analgesia, alleviated shivering in the parturient, and did not prolong the time of motor block or produced any side effects, which makes this dose appropriate for cesarean delivery [14].

Therefore, we tried to establish the day-case safety of 3 µg dexmedetomidine as an adjuvant intracavitary brachytherapy procedure. We observed the patients with intrathecal dexmedetomidine 3 µg stayed hemodynamically stable (without any bradycardia), with no sedation during and after the procedure. The majority of our patients did not require any rescue analgesia. Though the patient satisfaction scores in both groups were comparable, the patients receiving dexmedetomidine appeared way more comfortable and happier while mobilizing after the removal of applicators and at the time of discharge. This dose can be used safely in day-case intracavitary brachytherapy procedures without adverse effects. Dexmedetomidine 3 µg slowed motor recovery statistically, but practically, it did not seem to affect the patient's discharge from the hospital.

As further substantiated by our study, the 3 µg dexmedetomidine prolongs the absolute duration of spinal analgesia. The mean absolute duration of spinal analgesia in Group I was 406.82 ± 51.78 minutes as compared to 267 ± 16.23 minutes in Group II (p<.001). This is further supported by the results of Gupta et al., who did a comparative study of intrathecal dexmedetomidine 5 µg and fentanyl 25 µg as adjuvants to bupivacaine and found that intrathecal dexmedetomidine is associated with prolonged motor and sensory block, hemodynamic stability, and prolonged duration of analgesia, a reduced demand for rescue analgesics in 24 hours as compared to fentanyl. The duration of analgesia in their study was 251.7 ± 30.69 minutes in the dexmedetomidine group versus 168.96 ± 15.96 minutes in the fentanyl group [15].

The time taken for the onset of sensory effect at the T-10 level, two-segment regression from the T-10 dermatome, and hemodynamic vitals were comparable in both groups. There was no episode of significant bradycardia or excessive sedation in the dexmedetomidine group as compared to the fentanyl group. Patients were discharged home after fulfilling the following criteria: the patient was fully awake and responded to commands returned to pre-procedure status, the patient could sit in an upright position without signs and symptoms of orthostatic hypotension, the S-2 level dermatome of sensory regression, the patient could walk unaided, and the patient could micturate. The mean time to void and meet the discharge criteria in Group I was 355.71 ± 21.11 minutes and 258.43 ± 27.46 minutes in Group II. Haus et al. successfully used fentanyl 15 µg along with hyperbaric bupivacaine intrathecally in day-care brachytherapy for carcinoma of the cervix [16]. The mean time taken to discharge the patients from the hospital in the fentanyl 15 µg group was 240 minutes, which is quite similar to the findings of our study.

We did not note the exact time of removal of applicators after radiation treatment. This could have helped us know the precise duration of the complete procedure. This was the limitation of our study. There should be more research to find suitable alternatives for opioid-free anesthesia and analgesia. Going forward, there must be more studies to build evidence about the intrathecal use of dexmedetomidine 3 µg as an adjuvant to low-dose hyperbaric bupivacaine in other day-case surgeries. The research must be towards the use and comparison of other isomeric forms of bupivacaine with adjuvants like dexmedetomidine and fentanyl intrathecally. This may further reduce the motor blockade, alongside maintaining the beneficial effects of high-quality and prolonged analgesia.

## Conclusions

Adequate analgesia after a brachytherapy procedure is essential for quality discharge from the hospital. It improves comfort levels, maintains patient safety, and enhances overall patient satisfaction through the procedure. Both dexmedetomidine and fentanyl can be used safely as adjuvants to hyperbaric bupivacaine. Dexmedetomidine 3 µg dose as an adjuvant to low-dose hyperbaric bupivacaine can be used safely in day-case intracavitary brachytherapy procedures. It provides a prolonged duration of analgesia with no sedation as compared to fentanyl 15 µg with stable hemodynamics.

## References

[1] Toleska M, Dimitrovski A (2020). Is an opioid-free anaesthesia possible without using alpha-2 agonists?. Indian J Anaesth.

[2] Roy S, Loh HH (1996). Effects of opioids on the immune system. Neurochem Res.

[3] International Association for Ambulatory Surgery (IAAS) (2006). Day Surgery: Development and Practice.

[4] Kopp SL, Horlocker TT (2010). Regional anaesthesia in day-stay and short-stay surgery. Anaesthesia.

[5] Shah A, Patel I, Gandhi R (2013). Haemodynamic effects of intrathecal dexmedetomidine added to ropivacaine intraoperatively and for postoperative analgesia. Int J Basic Clin Pharmacol.

[6] Jarineshin H, Fekrat F, Kargar Kermanshah A (2016). Treatment of postoperative pain in pediatric operations: comparing the efficiency of bupivacaine, bupivacaine-dexmedetomidine and bupivacaine- fentanyl for caudal block. Anesth Pain Med.

[7] Mantz J, Josserand J, Hamada S (2011). Dexmedetomidine: new insights. Eur J Anaesthesiol.

[8] Piascik MT, Soltis EE, Piascik MM, Macmillan LB (1996). Alpha-adrenoceptors and vascular regulation: molecular, pharmacologic and clinical correlates. Pharmacol Ther.

[9] Gangadhar S, Gopal T, Sathyabhama Sathyabhama, Paramesh K (2012). Rapid emergence of day-care anaesthesia: a review. Indian J Anaesth.

[10] Rahimzadeh P, Faiz SH, Imani F, Derakhshan P, Amniati S (2018). Comparative addition of dexmedetomidine and fentanyl to intrathecal bupivacaine in orthopedic procedure in lower limbs. BMC Anesthesiol.

[11] Bromage PR (1965). A comparison of the hydrochloride and carbon dioxide salts of lidocaine and prilocaine in epidural analgesia. Acta Anaesthesiol Scand Suppl.

[12] Bajwa BS, Singh AP, Rekhi AK (2017). Comparison of intrathecal clonidine and fentanyl in hyperbaric bupivacaine for spinal anesthesia and postoperative analgesia in patients undergoing lower abdominal surgeries. Saudi J Anaesth.

[13] Nethra SS, Sathesha M, Dixit A, Dongare PA, Harsoor SS, Devikarani D (2015). Intrathecal dexmedetomidine as adjuvant for spinal anaesthesia for perianal ambulatory surgeries: a randomised double-blind controlled study. Indian J Anaesth.

[14] Bi YH, Wu JM, Zhang YZ, Zhang RQ (2020). Effect of different doses of intrathecal dexmedetomidine as an adjuvant combined with hyperbaric ropivacaine in patients undergoing cesarean section. Front Pharmacol.

[15] Gupta R, Bogra J, Verma R, Kohli M, Kushwaha JK, Kumar S (2011). Dexmedetomidine as an intrathecal adjuvant for postoperative analgesia. Indian J Anaesth.

[16] Haus NJ, Kambarami TC, Dyer RA (2013). Spinal anesthesia for brachytherapy for carcinoma of the cervix: a comparison of two dose regimens of hyperbaric bupivacaine. South Afr J Anaesth Analg.

